# Epidemiology and antifungal susceptibilities of clinically isolated *Aspergillus* species in South China

**DOI:** 10.1017/S095026882300167X

**Published:** 2023-10-17

**Authors:** Hazrat Bilal, Dongxing Zhang, Muhammad Shafiq, Muhammad Nadeem Khan, Canhua chen, Sabir Khan, Lin Cai, Rahat Ullah Khan, Haibin Hu, Yuebin Zeng

**Affiliations:** 1 Department of Dermatology, Second Affiliated Hospital of Shantou University Medical College, Shantou, China; 2 Department of Dermatology, Meizhou Dongshan Hospital, Meizhou, Guangdong Province, China; 3 Department of Dermatology, Meizhou People’s Hospital, Meizhou, Guangdong Province, China; 4 Research Institute of Clinical Pharmacy, Shantou University Medical College, Shantou, China; 5 Faculty of Biological Sciences, Department of Microbiology, Quaid-I-Azam University, Islamabad, Pakistan; 6 Clinical Laboratory, Meizhou People’s Hospital, Meizhou, Guangdong Province, China; 7 Institute of Microbiology Faculty of Veterinary and Animal Sciences, Gomal University, Dera Ismail Khan, Pakistan; 8 The First Clinical Medical College, Guangdong Medical University, Zhanjiang, China; 9 Department of Dermatology, West China Second University Hospital, Sichuan University, Chengdu, Sichuan, China

**Keywords:** antifungal susceptibilities, aspergillosis, clinical characteristics, epidemiology, South China

## Abstract

Aspergillosis is a rising concern worldwide; however, its prevalence is not well documented in China. This retrospective study determined *Aspergillus’s* epidemiology and antifungal susceptibilities at Meizhou People’s Hospital, South China. From 2017 to 2022, the demographic, clinical, and laboratory data about aspergillosis were collected from the hospital’s records and analysed using descriptive statistics, chi-square test, and ANOVA. Of 474 aspergillosis cases, *A. fumigatus* (75.32%) was the most common, followed by *A. niger* (9.92%), *A. flavus* (8.86%), and *A. terreus* (5.91%). A 5.94-fold increase in aspergillosis occurred during the study duration, with the highest cases reported from the intensive care unit (52.74%) – chronic pulmonary aspergillosis (79.1%) and isolated from sputum (62.93%). Only 38 (8.02%) patients used immunosuppressant drugs, while gastroenteritis (5.7%), haematologic malignancy (4.22%), and cardiovascular disease (4.22%) were the most prevalent underlying illnesses. In *A. fumigatus,* the wild-type (WT) isolates against amphotericin B (99.1%) were higher than triazoles (97–98%), whereas, in non-*fumigatus Aspergillus* species, the triazole (95–100%) WT proportion was greater than amphotericin B (91–95%). Additionally, there were significantly fewer WT *A. fumigatus* isolates for itraconazole and posaconazole in outpatients than inpatients. These findings may aid in better understanding and management of aspergillosis in the region.

## Introduction

Aspergillosis is a severe fungal infection caused by various *Aspergillus* species found in soil, rotting organic matter, and confined spaces. *Aspergillus fumigatus* is a frequently observed species that causes approximately 70–80% of human aspergillosis cases. Infections with other species, such as *Aspergillus niger*, *Aspergillus flavus*, and *Aspergillus terreus*, are also becoming more common, particularly in immunocompromised hosts [1]. It can affect several organs and manifest in various clinical forms, such as pulmonary, otoaspergillosis, endophthalmitis, systemic, and cutaneous aspergillosis. Pulmonary aspergillosis occurs in various clinical forms, such as invasive pulmonary aspergillosis (IPA), chronic pulmonary aspergillosis (CPA), bronchial colonisation, allergic bronchopulmonary aspergillosis, and severe asthma [2]. The global burden of aspergillosis is rising, posing a threat to healthcare systems worldwide. The prevalence of aspergillosis in China is, however, poorly documented, with only a few studies conducted in specific areas [3]. A study in 2017 reported global estimates of 3,000,000 cases of chronic pulmonary aspergillosis, and ~ 250,000 cases of invasive aspergillosis [4]. The immunocompromised hosts, such as those with haematological malignancies, solid organ transplants, and chronic lung diseases, and those exposed to environmental triggers such as dust and mould, are disproportionately affected by aspergillosis [5].

Aspergillosis is typically treated with triazole antifungal drugs such as voriconazole, itraconazole, isavuconazole, and posaconazole. Amphotericin B and caspofungin are significant antifungal agents against azole-resistant isolates. Caspofungin is typically not used alone but is given as a part of a multidrug antifungal regimen along with azoles [6]. However, the selection of a specific drug is determined by various factors, including the type of aspergillosis, infection severity, and the susceptibility profile of the concerned *Aspergillus* isolates [7]. Antifungal susceptibility testing is essential for selecting appropriate antifungal therapy because it identifies wild-type and non-wild types *Aspergillus* isolates against particular antifungal agents [8].

Antifungal susceptibility of *Aspergillus* isolates varies based on species types, geographical region, and local antifungal consumption patterns [9]. Recently, there has been an increase in antifungal non-wild-type *Aspergillus* isolates, especially for azole drugs, which are the primary therapy for aspergillosis [10]. Surveillance studies based on antifungal susceptibility patterns in *Aspergillus* species are critical in identifying emerging non-wild types isolates, directing the appropriate selection of an antifungal agent, and significantly impacting aspergillosis management and outcome [11].

This retrospective study aimed to analyse the epidemiology, risk factors, and antifungal susceptibilities of aspergillosis in a tertiary care hospital in the eastern Guangdong region of South China. The findings of this study will help healthcare officials manage aspergillosis and may have implications for the selection of appropriate antifungal agents and treatment strategies in the region.

## Methods

### Study design and setting

The current retrospective study was performed at a tertiary care hospital named Meizhou People’s Hospital in Meizhou, South China [12]. The study included all aspergillosis cases reported from January 2017 to December 2022. The ethical review committee of the hospital approved the study following the standards of the Helsinki Declaration (Reference number: 2021-C-106).

### Definitions

The aspergillosis cases reported in the current study were classified as CPA, IPA, otoaspergillosis, and cutaneous aspergillosis. Chronic and invasive pulmonary aspergilloses were diagnosed and classified based on the European Organization for Research and Treatment of Cancer (EORTC) and the Mycoses Study Group Education and Research Consortium (MSG/ERC) guidelines [13, 14]. Chronic pulmonary aspergillosis is a slowly progressing infection that affects immunocompromised patients or those with underlying lung illnesses. It is distinguished by cavities or lung fibrosis, positive *Aspergillus* serology, and symptoms lasting more than 3 months. Invasive pulmonary aspergillosis is a quickly progressing infection that primarily affects people who have significant immunosuppression or are critically unwell. It is distinguished by blood vessel invasion and spread to other organs, positive biomarkers such as galactomannan or PCR for *Aspergillus*, and symptoms lasting fewer than 3 months. Otoaspergillosis is an *Aspergillus* infection that affects the ear, frequently resulting in otitis externa or otomycosis. Cutaneous aspergillosis is a type of *Aspergillus* infection that affects the skin, resulting in skin lesions or ulcers [15].

### Data collection

The demographic and clinical data of all aspergillosis patients were collected from the hospital’s electronic medical record using a Microsoft Excel sheet (2021). The data included patients’ characteristics such as gender, age, seasonal infection period, patient type, reported department, sample source, infection type, and underlying patient status. Moreover, the laboratory record of *Aspergillus* species type and their antifungal susceptibility testing were also collected. Data analysis was confined to only the first positive culture for patients with repeated positive cultures for *Aspergillus* species.

In routine laboratory protocol, *Aspergillus* species are identified by combining colony morphology and matrix-assisted laser desorption/ionisation–time of flight mass spectrometry (MALDI-TOF MS) analysis (Bruker Daltonik, Bremen, Germany) [16]. The identification was carried out with the use of the Bruker library application Spectra (version 4.0.0.1, which contained 5627 entries) that came preinstalled on the Bruker Biotyper (version 3.1; Bruker.1). All the cases included in the current study were confirmed by the clinical characteristics and routine laboratory protocols such as microscopy, culture, serological test (*Aspergillus*-specific IgG antibodies and galactomannan (GM) test), and radiography (if needed). The current study only included *Aspergillus* infection patients; cases of colonisation were excluded. The sputum samples in the current study were expectorated sputum, in which the aforementioned methods confirmed the infection. Furthermore, the antifungal susceptibilities testing (AST) was performed against five drugs: amphotericin B, caspofungin, itraconazole, voriconazole, and posaconazole (Sigma Aldrich, St. Louis, MO, USA). The AST was performed following the standard protocol of broth microdilution methods. The *Candida krusei* ATCC 6258 and *Candida parapsilosis* ATCC 22019 were quality control strains. The AST results were interpreted according to the Clinical and Laboratory Standards Institute (CLSI M38-A3) guidelines [17]. The Minimum Inhibitory Concentration (MIC) for itraconazole, voriconazole, posaconazole, and amphotericin B was determined after 48 h as the lowest concentration preventing observable growth. The Minimum Effective Concentration (MEC) for caspofungin was determined as the lowest concentration that caused the growth of small, rounded, compact hyphal forms compared to the hyphal growth found in the growth control well at 24 h [18–20].

### Data analysis

Two researchers independently cross-checked all the information collected via Microsoft Excel to minimise possible errors. The quantitative findings were expressed as medians and interquartile ranges, while qualitative information was presented as absolute numbers and relative percentages. The characteristics of aspergillosis caused by various *Aspergillus* species (*A. fumigatus, A. niger, A. flavus*, and *A. terreus*) were analysed using the chi-square test for categorical variables and ANOVA for continuous variables. For AST data, the ranges of MICs, MECs, geometric mean (GM), and absolute numbers and percentages of MIC50/MEC50, MIC90/MEC90, wild-type (WT), and non-wild-type (NWT) isolates were quantified for each of the *Aspergillus* species types. Furthermore, the number of WT isolates against different antifungal agents recovered from inpatients and outpatients was statistically analysed using the chi-square test. The *p*-values less than 0.05 were considered statistically significant. GraphPad Prism *v.8.0.2* was used for statistical analysis and visualisation of the data.

## Results

### Incidence of aspergillosis

**Figure 1.**
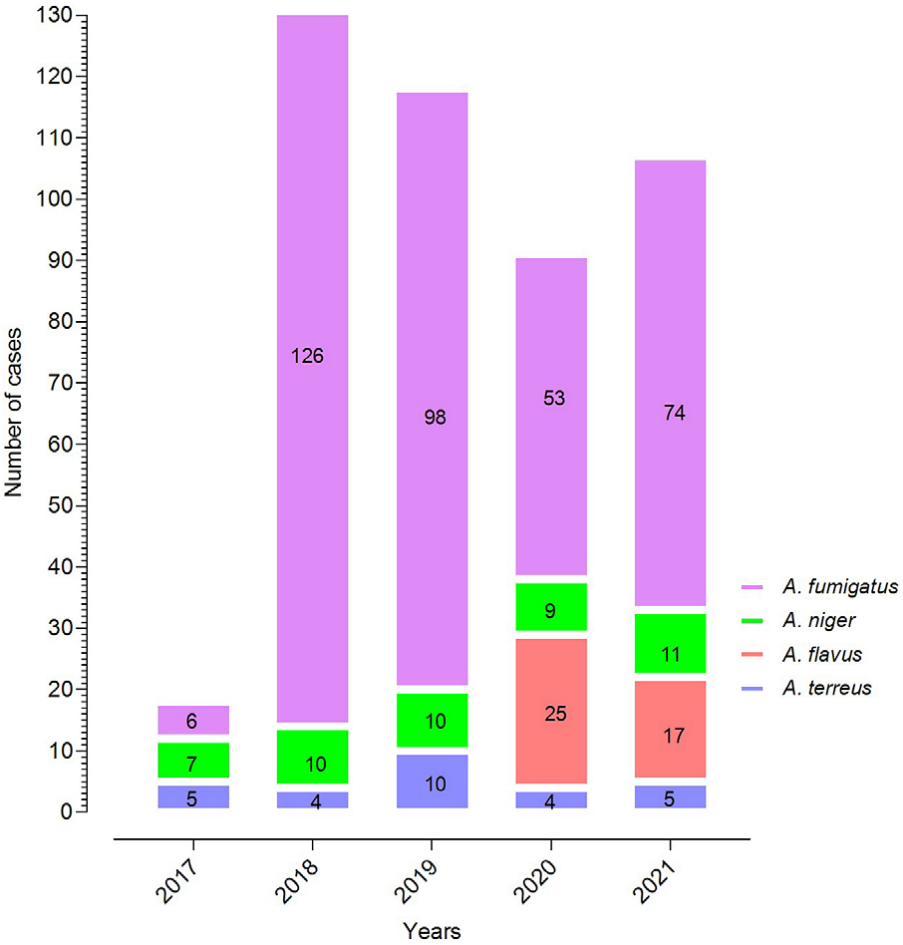
The incidence of Aspergillus species reported yearly from 2017 to 2021.

In the current study, a total of 474 cases of aspergillosis were reported, in which a high number of cases were caused by *Aspergillus* section *Fumigati* n = 357,), followed by *Aspergillus* section *Nigri* n = 47), *Aspergillus* section *Flavi* n = 42), and *Aspergillus* section *Terrei* n = 28). The high number of cases were reported in the year 2018 (n = 140,), followed by 2019 (n = 118,), while only 18 cases were reported in 2017. The fluctuation in number of cases each year was reported; however, a 5.94-fold increase in aspergillosis was observed from 2017 to 2021. The complete depiction of different *Aspergillus* species reported each year is presented in (Figure 1).

**Figure 2.**
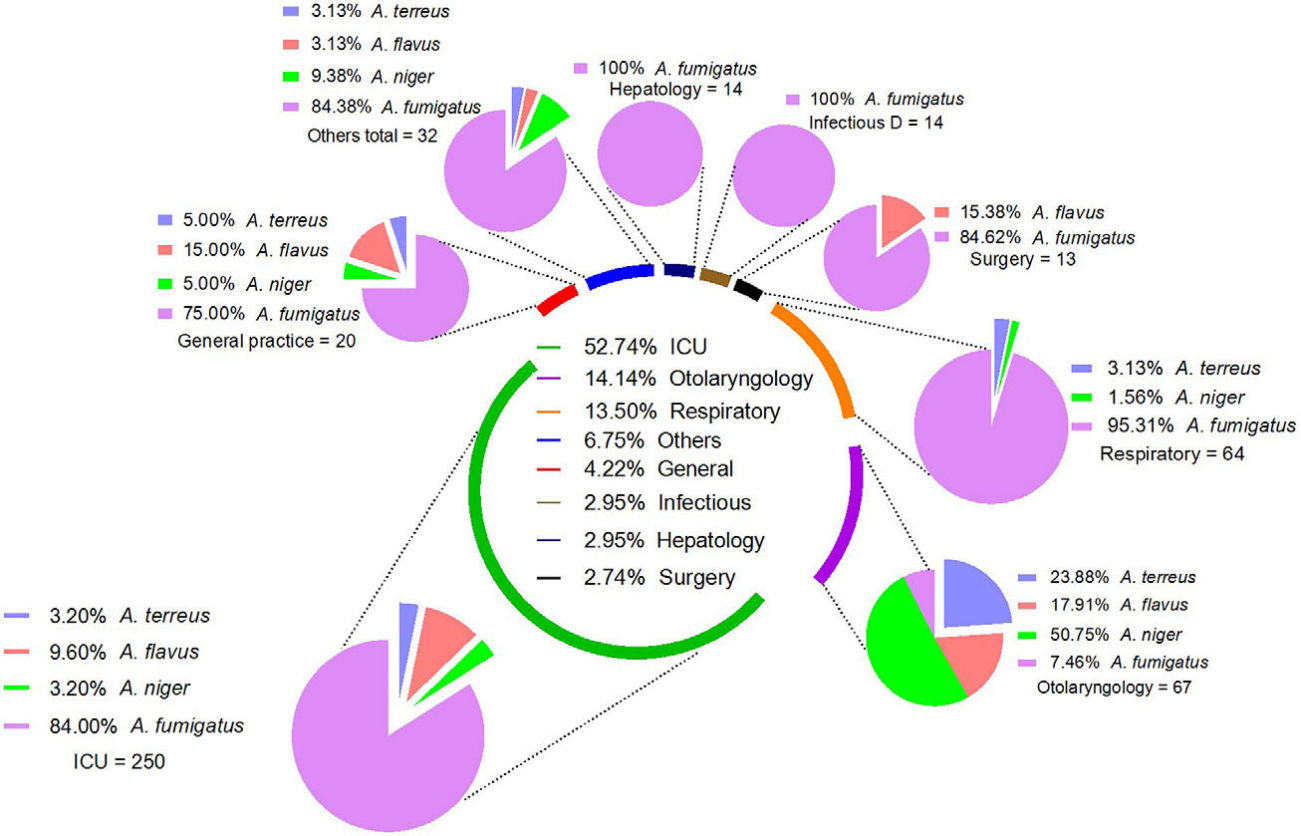
Aspergillosis cases reported from different departments of the hospital. Each pie chart in the surrounding shows the incidence of Aspergillus species from each department. In the department, others represent 7 cases each from cardiology and urology, 4 cases each from gastroenterology and Chinese medicine, 2 cases each from neurology, immunology, endocrinology, and haematology, and 1 case each from the dermatology and orthopaedics department.

Among various departments of the hospital, a high number of cases were reported from the intensive care unit (ICU) (n = 250, 52.74%), followed by the surgical department, from where a total of 13 (2.74%) cases were reported. A high number of cases from each department were caused by *A. fumigatus*, except otolaryngology, in which 50.75% (n = 34) cases were *A. niger* among the 67 reported cases. The percentages of aspergillosis cases from various departments of the hospital and different *Aspergillus* species from each department are presented in (Figure 2).

**Figure 3.**
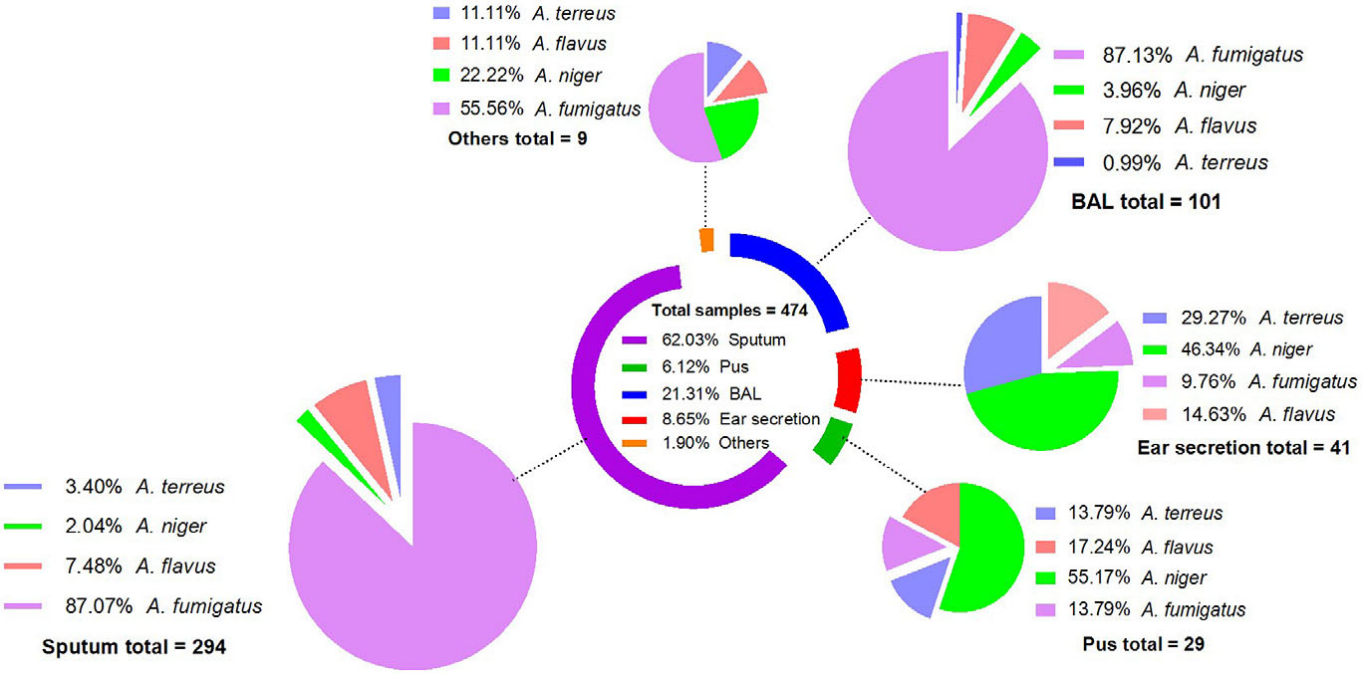
Aspergillus species isolated from different sample types. Each pie chart shows the incidence of Aspergillus species from each sample type. In the sample types, Others represent two samples each for wound and pleural effusion and one for catheter, nail, tissue, peritoneal fluid, and renal drainage.

Among the sample types, sputum had the highest frequency of *Aspergillus* isolates (n = 294, 62.03%), followed by bronchoalveolar lavage fluid (BALF) with 101 (21.31%) total cases. From both the samples, *A. fumigatus* was frequently isolated. Ear secretion and pus samples had lower frequencies, with 41 (8.65%) and 29 (6.12%) samples, respectively. The *A. niger* was the most frequently isolated species in both sample types, with 19 (46.34%) and 16 (55.17%) samples, respectively. The distribution of *Aspergillus* species isolated from different sample types is shown in Figure 3.

### Characteristics of aspergillosis

The demographic and clinical characteristics of aspergillosis caused by various *Aspergillus* species are presented in Table 1. Based on gender, a higher number of cases were reported in the male population (n = 315, 66.46%), while only 159 (33.36%) cases were reported among females. The same trend occurred for all species except *A. niger*, in which most cases (n = 29, 61.7%) were reported from the female population. Most cases were reported in senior adults with a median age of 65 years and an interquartile range of 52–76. Comparatively, the *A. niger* cases were reported in younger ages with a median age of 44 years (IQR; 35–56), followed by *A. terreus* (57 years, 1QR; 37–76).

**Table 1.** Baseline characteristics of *Aspergillus* species

Variables	Total (474, 100%)	*A. fumigatus* (357, 75.32%)	*A. niger* (47, 9.92%)	*A. flavus* (42, 8.86%)	*A. terreus* (28, 5.91%)	X2, df	*p*-values
**Gender**							
Male	315 (66.46)	246 (68.91)	18 (38.3)	32 (76.2)	19 (67.86)	19.49, 3	0.0002
Median age (IQR)	65 (52–76)	67 (56–78)	44 (35–56)	64 (53–75)	57 (37–76)	0.1666	<0.0001
Age groups							
2 to 17	5 (1.06)	0 (0)	5 (10.64)	0 (0)	0 (0)	45.91, 3	<0.0001
18 to 60	173 (36.5)	106 (29.7)	33 (70.2)	20 (47.6)	14 (50)	34.63, 3	<0.0001
61 to 80	233 (49.1)	194 (54.3)	9 (19.2)	18 (42.9)	12 (42.8)	21.89, 3	<0.0001
>80	63 (13.3)	57 (15.97)	0 (0)	4 (9.53)	2 (7.14)	10.86, 3	0.0125
Seasons							
Winter	162 (34.18)	134 (37.54)	8 (17.03)	9 (21.43)	11 (39.29)	11.30, 3	0.0102
Spring	110 (23.21)	84 (23.53)	7 (14.9)	9 (21.43)	10 (35.71)	4.376, 3	0.2236
Summer	93 (19.63)	67 (18.77)	13 (27.66)	10 (23.81)	3 (10.71)	3.966, 3	0.2651
Autumn	109 (23)	72 (20.17)	19 (40.43)	14 (33.34)	4 (14.29)	13.41, 3	0.0038
Patients’ types							
Inpatients	380 (80.17)	317 (88.8)	20 (42.56)	27 (64.29)	16 (57.14)	74.54, 3	<0.0001
Outpatients	94 (19.84)	40 (11.21)	27 (57.45)	15 (35.72)	12 (42.86)	74.54, 3	<0.0001
Infection types							
Chronic pulmonary aspergillosis	375 (79.11)	319 (89.36)	17 (36.17)	24 (57.14)	15 (53.57)	98.44, 3	<0.0001
Invasive pulmonary aspergillosis	37 (7.80)	28 (7.84)	0 (0)	8 (19.05)	1 (3.57)	12.05, 3	0.0072
Oto-aspergillosis	56 (11.82)	5 (1.41)	29 (61.71)	10 (23.81)	12 (42.86)	181.1, 3	<0.0001
cutaneous aspergillosis	6 (1.27)	5 (1.41)	1 (2.13)	0 (0)	0 (0)	1.229, 3	0.7461
Underlying pulmonary status							
COPD	58 (12.27)	48 (13.44)	0 (0)	5 (11.90)	5 (17.86)	7.867, 3	0.0488
Bronchiectasis	43 (9.07)	34 (9.52)	2 (4.25)	4 (9.52)	3 (10.71)	1.512, 3	0.6794
Asthma	39 (8.23)	29 (8.12)	0 (0)	6 (14.29)	4 (14.28)	7.621, 3	0.0545
Pneumoniae	31 (6.54)	29 (8.12)	0 (0)	2 (4.76)	0 (0)	6.929, 3	0.0741
Respiratory failure	13 (2.74)	9 (2.52)	1 (2.13)	3 (7.14)	0 (0)	3.971, 3	0.2646
ABPA	7 (1.48)	7 (1.96)	0 (0)	0 (0)	0 (0)	2.329, 3	0.507
Tuberculosis	5 (1.06)	5 (1.41)	0 (0)	0 (0)	0 (0)	1.656, 3	0.6467
Underlying systematic disorders							
Gastrointestinal disorders	27 (5.7)	20 (5.61)	2 (4.26)	5 (11.91)	0 (0)	4.893, 3	0.1798
Renal failure	6 (1.27)	5 (1.41)	0 (0)	0 (0)	1 (3.57)	2.384, 3	0.4967
Hepatic disorders	12 (2.54)	9 (2.53)	1 (2.13)	1 (2.39)	1 (3.57)	0.1578, 3	0.9841
Haematologic malignancy	20 (4.22)	20 (5.61)	0 (0)	0 (0)	0 (0)	6.843, 3	0.0771
UTI	5 (1.06)	3 (0.85)	0 (0)	2 (4.77)	0 (0)	6.487, 3	0.0902
Solid Tumor	15 (3.17)	13 (3.65)	0 (0)	1 (2.39)	1 (3.57)	1.900, 3	0.5934
CNS disorders	13 (2.75)	13 (3.65)	0 (0)	0 (0)	0 (0)	4.381, 3	0.2232
Diabetes	12 (2.54)	7 (1.97)	1 (2.13)	3 (7.15)	1 (3.57)	4.244, 3	0.2362
cardiovascular disease	20 (4.22)	10 (2.81)	4 (8.52)	4 (9.53)	2 (7.14)	7.435, 3	0.0593
Hypertension	5 (1.06)	2 (0.57)	0 (0)	0 (0)	3 (10.71)	26.82, 3	<0.0001
Osteopathy	4 (0.85)	3 (0.85)	1 (2.13)	0 (0)	0 (0)	1.522, 3	0.6773
No underlying disorder reported	139 (29.32)	91 (25.49)	35 (74.47)	6 (14.29)	7 (25)	53.58, 3	<0.0001
Immunosuppressants drugs used	38 (8.02)	31 (8.69)	2 (4.26)	4 (9.53)	1 (3.57)	1.997, 3	0.5731

Abbreviations: X^2^ = chi-square, df = degree of freedom, *p*-value = probability value, IQR = Inter quartile range, COPD = Chronic Obstructive Pulmonary Disease, ABPA = Allergic Bronchopulmonary Aspergillosis, UTI = Urinary tract Infection.

The distribution of *Aspergillus* species among various age groups revealed statistically significant differences (*p* < 0.05). *A. niger* was more common in younger age groups (under 50 years), whereas *A. fumigatus* was more common in older age groups (over 50 years). Only five cases were reported in the age group 2–17 years, and all were *A. niger.* *A. flavus* was highly prevented in age groups older than 50, and thirteen (30.96%) cases were reported in age groups 51 to 60. These findings suggest that age may influence the prevalence of different *Aspergillus* species, emphasising the importance of considering age as a factor in understanding the epidemiology of aspergillosis.

Seasonally, the distribution of *Aspergillus* species varies significantly. During winter, *A. fumigatus* was the most prevalent species, whereas there was no significant difference in species distribution during spring and summer. However, *A. niger* was the most common species in autumn, with a statistically significant difference from other species (*p* = 0.0038). This suggests that seasonal environmental factors may influence the prevalence of *Aspergillus* species.

Most cases were reported among hospitalised patients (n = 380, 80.17%), while only 94 (19.84%) cases were reported from outpatients. The distribution of *Aspergillus* species based on patient type (inpatients vs. outpatients) was statistically significantly correlated (*p* < 0.0001). *A. fumigatus* (88.8%) was more frequently detected in hospitalised patients, whereas *A. niger* (57.45%) was more prevalent in outpatients.

The clinical and laboratory data about invasive and chronic aspergillosis are presented in Table S1 (supplementary file). Among the clinical manifestation, cough was the most commonly reported, with 85.07% in CPA patients and 72.79% in IPA patients. Among the others, fever was reported in high proportion in IPA (67.57%), while hemoptysis in CPA (54.4%) patients. Among the imaging manifestation, consolidation was reported in a high proportion in IPA patients (62.16%), while cavitation was reported more in CPA patients (64%). In the cases of CPA, most *Aspergillus* species were isolated from sputum culture (77.33%), while in IPA, most species were isolated from BALF culture (81.08%). The serum GM was detected in 11.2% of CPA patients and 59.46% of IPA patients, while BALF GM was found in 16.8% of CPA patients and 35.13% of IPA patients. The *Aspergillus*-specific antibodies were reported in 35.13% of IPA patients and 39.46% of CPA patients.

### Antifungal susceptibilities profiles

The antifungal susceptibilities profiles of *Aspergillus* species are summarised in Table 2. Amphotericin B had relatively high MIC values against all species ranging from 0.125 to 1 for MIC50 and 0.25 to 4 for MIC90. *A. niger* had the highest percentage of amphotericin B NWT isolates (4/47, 8.52%), followed by *A. terreus* (2/28, 7.15%), *A. flavus* (2/42, 4.77%), and *A. fumigatus* (3/357, 0.85%). Caspofungin had lower MEC values and a 100% WT percentage against *A. fumigatus* and *A. terreus.* In contrast, the percentages of NWT isolates in *A. niger* and *A. flavus* were 2/47 (4.26%) and 4/42 (9.53%), respectively. Among the tested azoles, posaconazole and voriconazole have lower MIC than itraconazole. *A. niger*, *A. flavus*, and *A. terreus* all showed 100% susceptibility to voriconazole. Similarly, *A. flavus* and *A. terreus* were 100% wild types for posaconazole. For itraconazole, a high number of NWT isolates were reported in the case of *A. terreus* (2/28, 7.15%), followed by *A. niger* (2/47, 4.26%), *A. fumigatus* (10/357, 2.81%), and *A. flavus* (1/42, 2.39%). Among the examined isolates, 5 *A. fumigatus*, 2 *A. niger*, and 1 *A. flavus* were NWT for more than one antifungal agent.

**Table 2.** Antifungal susceptibilities profiles of *Aspergillus* species detected in the current study

Antifungal agents	MIC/MEC values^a^											MIC/MEC ranges	MIC50/MEC50	MIC90/,MEC90		GM	ECV	WT (*n*, (%))	NWT (*n*, (%))
	0.015	0.03	0.06	0.125	0.25	0.5	1	2	4	8	16								
*A. fumigatus* (357)																			
Amphotericin B		2	1	8	4	15	182	142	3			0.03–4	1	2	1.19		2	354 (99.1)	3 (0.85)
Caspofungin	23	40	148	77	69							0.015–0.25	0.06	0.25	0.08		0.5	357 (100)	0 (0)
Itraconazole	2	8	12	40	102	93	90	1	3	6		0.015–8	0.5	1	0.39		1	347 (97.2)	10 (2.81)
Posaconazole		13	72	170	60	31	1	4	2		4	0.03–16	0.125	0.5	0.15		0.5	350 (98.04)	7 (1.97)
Voriconazole			3	9	186	81	71	2	1	4		0.06–8	0.25	1	0.4		1	350 (98.04)	7 (1.97)
*A. niger* (47)																			
Amphotericin B				17	9	13	4		3	1		0.125–8	0.25	1	0.35		2	43 (91.49)	4 (8.52)
Caspofungin	9	16	10	8	2	1	1					0.015–1	0.03	0.125	0.05		0.25	45 (95.75)	2 (4.26)
Itraconazole		1	4	1	4	15	13	6	1	2		0.03–8	0.5	2	0.62		4	45 (95.75)	2 (4.26)
Posaconazole			3	4	11	13	7	6	2	1		0.06–8	0.5	2	0.51		2	44 (93.62)	3 (6.39)
Voriconazole		1	3	4	13	23	3					0.03–1	0.5	0.5	0.32		2	47 (100)	0 (0)
*A. flavus* (42)																			
Amphotericin B			1	3	5	10	9	7	5	2		0.06–8	1	4	0.85		4	40 (95.24)	2 (4.77)
Caspofungin	3	1	2	5	11	7	9	3	1			0.015–4	0.25	1	0.31		0.5	38 (90.48)	4 (9.53)
Itraconazole	5	7	3	14	9	3	1					0.015–1	0.125	0.25	0.1		1	41 (97.62)	1 (2.39)
Posaconazole		4	8	13	17							0.03–0.25	0.125	0.25	0.13		0.5	42 (100)	0 (0)
Voriconazole			4	6	9	12	7	4				0.06–2	0.5	1	0.38		2	42 (100)	0 (0)
*A. terreus* (28)																			
Amphotericin B						1	2	13	10	2		0.5–8	2	4	2.57		4	26 (92.86)	2 (7.15)
Caspofungin	13	4	7	2	2							0.015–0.25	0.03	0.125	0.04		0.25	28 (100)	0 (0)
Itraconazole	3		1	2	3	9	8		1	1		0.015–8	0.5	1	0.39		2	26 (92.86)	2 (7.15)
Posaconazole	1	8	9	7	3							0.015–0.26	0.06	0.25	0.07		1	28 (100)	0 (0)
Voriconazole		1	2	2	14	7	2					0.03–1	0.25	0.5	0.27		2	28 (100)	0 (0)

Abbreviations: MIC = Minimum inhibitory concentration, MEC = Minimum effective concentration, GM = Geometric mean, ECV = Epidemiological cutoff values, WT = Wild types, NWT = non-wild types.

a For all drugs, MIC was determined except caspofungin, for which MEC was found.

**Figure 4.**
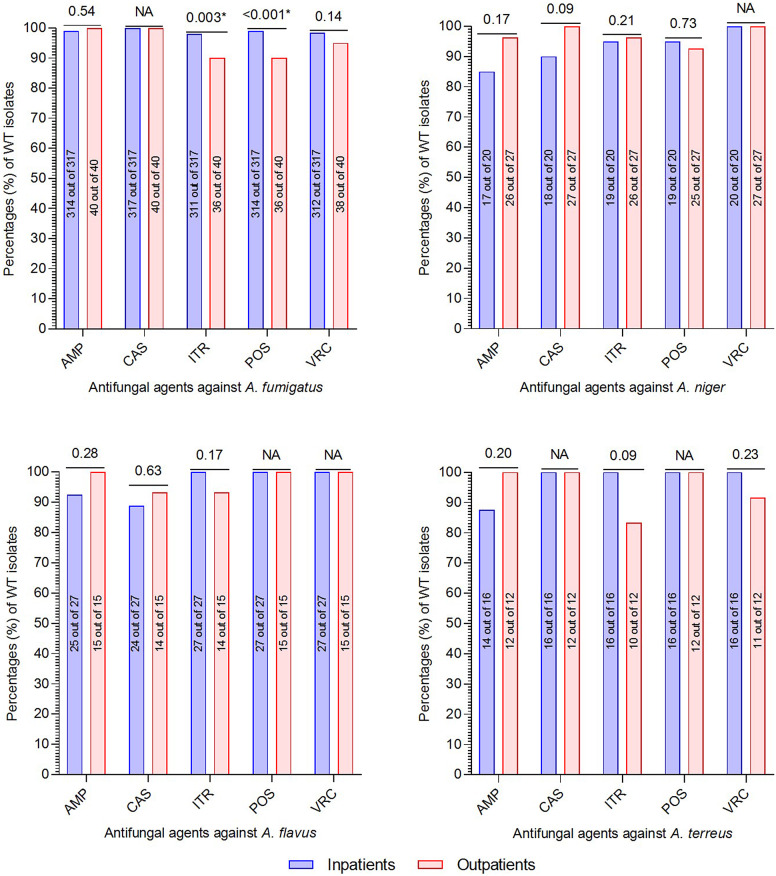
Comparatively analysis of wild-type isolates recovered from outpatients and inpatients. On the top of the bars are *p*-values derived by the chi-square test. NA; not applicable, AMP; amphotericin B, CAS; caspofungin, ITR; itraconazole, POS; posaconazole, VRC; voriconazole.

Furthermore, we compared the WT isolates reported from inpatients and outpatients to determine the relative difference in susceptibilities between the two groups. Interestingly, we noted that the percentages of WT isolates in the case of amphotericin B and caspofungin were comparatively lower for inpatients than outpatients. On the other hand, for azole drugs, the percentages of WT isolates were comparatively higher for inpatients than outpatients. However, the difference was only statistically significant (*p* < 0.05) in the case of itraconazole and posaconazole against *A. fumigatus*, while all others were statistically insignificant. The complete depiction of the comparative analysis of WT isolates in inpatients and outpatients is presented in Figure 4.

### Discussion

The current retrospective study analyses the incidence of *Aspergillus* species, as well as the demographic and clinical characteristics associated with them, in a tertiary care hospital in South China. A 5.94-fold increase in aspergillosis was reported during the study duration. The increasing trend is inconsistent with previous reports from China [1, 21]. Several factors, such as improving diagnostic capabilities, awareness in healthcare professionals, and changes in patients’ population and risk factors, contributed to the increase in aspergillosis [22, 23]. Furthermore, a significant increase in aspergillosis incidence, particularly for *A. fumigatus*, was reported between 2017 and 2018. In 2017, the international guidelines for diagnosing aspergillosis were less practical as those were recently published at that time (2016) [14]. Histopathology and tissue cultures were considered gold-standard techniques for diagnosing aspergillosis at earlier times [24]. However, health professionals were later aware of international guidelines, which might be the reason for reporting the high number of *A. fumigatus* in 2018 and onward. The *A. fumigatus* were reported in high proportion, comprising 75.32% of the total isolates. The *A. fumigatus* is a worldwide highly reported *Aspergillus* species, previously reported in high proportion from China, USA, Europe, Africa, and Australia. The most frequent reporting of *A. fumigatus* might be due to its opportunistic pathogenicity, ubiquity, fast growth, and competitive advantage [25]. The *A. niger* was the second most commonly isolated species in the current study, just like previously reported from Korea [26]. However, some other studies from Brazil and Australia reported a high proportion of *A. flavus* and *A. terreus* [27, 28]. Different trends in the prevalence of *Aspergillus* species indicate that it might be due to geographically oriented variation [1]. The *A. flavus* were not reported in the years 2017 to 2019, while in 2020 and 2021, a total of 25 and 17 cases were reported, respectively. *A. flavus* is a prevalent infectious species in Asia; nevertheless, its occurrence is comparatively smaller and more geographically limited when compared to *A. fumigatus* [29]. The non-detection of *A. flavus* in our investigation from 2017 to 2019 may indicate geographical or temporal disparities in the occurrence of this species, potentially driven by environmental variables such as temperature, humidity, and agricultural practices [30]. However, the absence noticed of *A. flavus* within a given time period may be a chance occurrence. It is suggested that further investigation should be conducted to explore the underlying factors contributing to the temporal fluctuations of *A. flavus* in the specified area. This can be achieved by using a more extensive sampling approach over an extended timeframe, in order to enhance our comprehension of the species’ ecological dynamics. Among the hospital’s various departments, more than half of the total cases (52.74%) were reported from the ICU department in the present study. Several factors favour the development of aspergillosis in ICU patients, in which critical underlying status, prolonged corticosteroid treatment, and systemic diseases requiring immunosuppressive therapy are prominent [31]. Early diagnosis, swift antifungal treatment, strict infection control, optimisation of the immune status, and ongoing education and surveillance are needed to overcome ICU aspergillosis [32].

Regarding the sample sources, sputum was the most commonly reported specimen, followed by BAL fluids having *A. fumigatus* isolated in high proportion from both specimens. The current study’s finding is similar to the previously reported 20-year retrospective study from China, indicating the endemic nature of *A. fumigatus* as a respiratory tract pathogen in the region [1]. The *A. niger* was isolated in high proportion from pus and ear secretion specimens. The association between *A. niger* and ear infection has been well documented in previous studies, which reported that almost half of otomycosis is caused by *A. niger.* This might be due to *A. niger* preference for the external auditory canal and its ability to produce a large number of conidia [33].

The distribution of *Aspergillus* species based on gender is always controversial. In the current study, *A. fumigatus*, *A. flavus*, and *A. terreus* were found in large proportions in males, while *A. niger* was found in high percentages in females. Smoking habits and occupational exposure to these species are frequently reported as likely causes of pulmonary aspergillosis among the male population [34]. While *A. niger* was primarily reported in association with otomycosis, the high incidence of this fungus in the female population and younger age group might be attributed to their propensity for using cosmetics [35]. The precise mechanism linking cosmetic usage to *A. niger* infections was not investigated in our study or other reported studies; nonetheless, we hypothesised that cosmetics, particularly those used in the ear region, could transfer fungal spores or establish an environment favourable to fungal growth. The use of infected or expired cosmetic items, poor hygiene, or excessive moisture in the ear due to cosmetic use may all lead to a rise in *A. niger* infections. Furthermore, there is currently no robust, large-scale investigation that can conclusively determine the impact of gender on the development of aspergillosis. Therefore, further research is needed to better understand the role of gender in aspergillosis risk factors.

Regarding age, most cases were reported in the senior adult age group, with a median age of 65 years (IQR; 52–76). The present investigation found that *A. fumigatus*, *A. flavus*, and *A. terreus* exhibit a high prevalence among individuals aged 50 and above. Additionally, it was observed that these species are predominantly linked to cases of pulmonary aspergillosis, particularly among patients in the ICU who have significant underlying pulmonary and systemic disorders. The greater vulnerability of older people to aspergillosis may be attributed to their impaired immune system and the presence of significant underlying co-morbidities. [36]. Unlike the other species in the current study, *A. niger* was reported comparatively high in the younger age group (< 50 years). Occupational dust exposure and poor hygiene, such as using non-sterile ear-cleaning instruments, can cause *A. niger* growth in the external auditory canal and high morbidity rates in this age group [37]. Additionally, in the paediatric population (age group 2 to 17 years), only five cases were reported, all attributed to *A. niger.* We speculate that the high incidence of *A. niger* in females may also serve as a potential explanation for these infections in children. It is imaginable that the close attachment between children and their mothers could be a contributing factor [38]. Further investigation is warranted to explore the potential link between maternal exposure and *A. niger* infections in paediatric patients. No prominent differences in *Aspergillus* incidence based on season were reported in the current study. Similar to our findings, previously published studies from Serbia and Spain reported no association of specific seasons with aspergillosis [39, 40]. However, the prevalence was a little higher in winter (34.18%) than in spring (23.21%), summer (19.63%), and autumn (23%). This might be due to the increased indoor activities and cold and damp winter environments [41]. More cases were reported in inpatients (80.17%) than outpatients (19.84%). The opportunistic nature of the *Aspergillus* species, immunocompromised status, underlying health issues, exposure to invasive medical procedures, and antifungal use increased the risk of aspergillosis in inpatients [42]. Among the patients’ underlying issues were a higher proportion of immunosuppressant drugs used (8.02%), followed by gastrointestinal disorder (5.7%), haematological malignancy (4.22%), and cardiovascular disorders (4.22%). These risk factors are worldwide reported in association with aspergillosis [43–45]. However, none of the underlying statuses was statistically significant to *Aspergillus species* except hypertension, which occurs in a high proportion (10.71%) in *A. terreus* infection (*p* < 0.0001). There is currently no documented study that links *A. terreus* to hypertension. Hypertension is a complicated medical condition influenced by various factors, including age, family history, excessive alcohol consumption, physical inactivity, obesity, smoking, poor diet, diabetes, kidney disease, and immune system dysfunction [46]. However, the association between hypertension and *A. terreus* remains unknown and will need to be investigated in future studies.

Regarding the antifungal susceptibility testing, we found that in the case of *A. fumigatus,* the proportion of WT isolates against amphotericin (99.1%) was higher than triazoles (97–98%). Similar results were reported in previously published literature from various regions of China and Europe. The high proportion of WT isolates against amphotericin B might be because of its fungicidal and limited use due to its nephrotoxicity [47]. Contrarily, triazoles are first-line therapy against aspergillosis, leading to the emergence of azole non-wild types isolates [48, 49]. Conversely, in non-*fumigatus Aspergillus* species, the triazole (95–100%) WT proportion was higher than amphotericin B (91–95%). These findings are in line with previous research [1, 50]. However, based on these findings, it is hard to classify triazoles as effective antimicrobials against non-*fumigatus* species theoretically. One factor to consider is the small number of non-*fumigatus* strains included in the current study compared to *A. fumigatus* isolates. Additional large-scale molecular studies focusing on triazole susceptibility in non-*fumigatus* isolates are required to confirm this hypothesis. Among the triazoles, the proportion of NWT isolates against itraconazole was high compared to posaconazole and voriconazole. It might be linked to its wide use as a first-line and alternative therapy for chronic pulmonary aspergillosis and allergic bronchopulmonary aspergillosis, respectively. Furthermore, by comparative analysis of AST profiles for inpatients and outpatients, it was noted that triazole susceptibilities were comparatively lower for outpatients than inpatients. The triazoles non-wild types isolates in outpatients might be attributed to the high use of triazoles as an agricultural antifungal agent [51]. A study reported that agriculture’s entire quantity of azoles accounted for over one-third of all antifungal agents used in China [52]. This high use of azoles causes the emergence of azole-non-wild-type environmental *Aspergillus* species, which can infect humans and other animals [53]. Continuous surveillance studies under the One-Health approach are required to monitor the emergence of non-wild types *Aspergillus* species.

The limitation of the current study is its retrospective nature, so some data might not be available for analysis; for example, all the species were not accessible for molecular identification, which have a higher level of specificity in identifying fungal species. This study is based on a single centre in South China with a limited sample size, especially for non-*fumigatus* isolates. Therefore, the results might not be generalisable for other regions in China. Furthermore, we did not have access to data on antifungal therapy prior to the isolation of the *Aspergillus* species for the patients included in our study. As a result, we could not investigate the effect of prior antifungal medication on resistance development. However, the current study’s findings might help health officials manage aspergillosis in the regions.

Future prospective studies based on molecular epidemiology and antifungal resistance mechanisms from multi-centres are required. The researchers need to specifically target the mutation determinant of the CYP51A gene and its linkage with azole NWT isolates. Moreover, it is necessary to determine the correlation between prior antifungal therapy and the development of resistance in fungal isolates.

## Conclusion

The present study reported 474 aspergillosis cases, with a high proportion of cases caused by *A. fumigatus.* Among the non-*fumigatus* cases, *A. niger* was reported in high proportion, followed by *A. flavus.* A high number of cases were reported from the ICU, indicating the immunocompromised status of the patients. Chronic pulmonary aspergillosis was reported in high proportion, showing that *Aspergillus* species are mainly involved in respiratory tract pathogenicity. Amphotericin B appears to be the most active antifungal agent against *A. fumigatus*, while for non-*fumigatus* isolates, the triazole’s MICs were lower than amphotericin B, which warrants further large-scale research confirmation. The *Aspergillus* species reported from outpatients have lower MIC/MEC to amphotericin B and caspofungin than triazoles, indicating that the agricultural use of triazole led to this emergence of non-wild types isolates. Further extensive molecular-based surveillance studies under the umbrella of One-Health approaches are required for the continuous monitoring of *Aspergillus* species, which will help manage aspergillosis.

## Supporting information

Bilal et al. supplementary material Bilal et al. supplementary material

## Data Availability

All the data are presented in the manuscript; any raw data can be available by request to the first author (email; bilal.microbiologist@yahoo.com).

## Supplementary material

For supplementary material accompanying this paper visit http://doi.org/10.1017/S095026882300167X. click here to view supplementary material
