# Supplementary material for: Epidemiology and antifungal susceptibilities of clinically isolated Aspergillus species in South China
Source: Epidemiol Infect. 2023 Oct 17;151:e184. doi: 10.1017/S095026882300167X (PMC10644062; doi:10.1017/S095026882300167X)
Supplement: Bilal et al. supplementary material [file S095026882300167Xsup001.pdf]

Table S 1: Characteristics of Chronic and invasive pulmonary aspergillosis.

**Variable**	**Total (*n* = 412)**	**Chronic pulmonary aspergillosis (*n*= 375)**	**Invasive pulmonary aspergillosis (*n* = 37)**	**Z value**	**p-values**
**Clinical manifestation**					
Cough	346	319 (85.07%)	27 (72.97%)	1.913	0.0557
Hemoptysis	216	204 (54.4%)	12 (32.43%)	2.553	0.0107
Dyspnea	115	98 (26.13%)	17 (45.95%)	2.563	0.0104
Fever	137	112 (29.86%)	25 (67.57%)	4.644	<0.0001
Chest pain	65	56 (14.93%)	9 (24.32%)	1.495	0.1349
Fatigue	60	43 (11.47%)	17 (45.95%)	5.673	<0.0001
Nigh sweat	37	33 (8.8%)	4 (10.81%)	0.4082	0.6832
**Underlying systematic disorders**					
Gastrointestinal disorders	27	25 (6.67%)	2 (5.4%)	0.3661	0.7143
Renal failure	6	1 (0.27%)	5 (13.51%)	6.257	<0.0001
Hepatic disorders	12	8 (2.13%)	4 (10.81%)	2.888	0.0039
Hematologic malignancy	20	18 (4.8%)	2 (5.4%)	0.09585	0.9236
UTI	5	4 (1.07%)	1 (2.7%)	0.8185	0.4131
Solid Tumor	15	12 (3.2%)	3 (8.1%)	1.436	0.1509
CNS disorders	13	13 (3.47%)	0 (0)	1.180	0.2378
Diabetes	12	8 (2.13%)	4 (10.81%)	2.888	0.0039
cardiovascular disease	20	19 (5.07%)	1 (2.7%)	0.6909	0.4896
Hypertension	5	5 (1.33%)	0 (0)	0.7245	0.4688
Osteopathy	4	2 (0.53%)	2 (5.4%)	2.799	0.0051
**Underlying pulmonary status**					
COPD	58	52 (13.87%)	6 (16.22%)	0.2697	0.7874
Bronchiectasis	43	42 (11.2%)	1 (2.7%)	1.683	0.0924
Asthma	39	38 (10.13%)	1 (2.7%)	1.540	0.1236
Pneumoniae	31	31 (8.26%)	0 (0)	1.867	0.0618
Respiratory failure	13	8 (2.13%)	5 (13.51%)	3.654	0.0003
ABPA	7	7 (1.87%)	0 (0)	0.8594	0.3901
Tuberculosis	5	5 (1.33%)	0 (0)	0.7245	0.4688
**Imaging manifestation**					
Consolidation	64	41 (10.93%)	23 (62.16%)	8.207	<0.0001
Cavitation	245	240 (64%)	5 (13.51%)	5.968	<0.0001
Nodules	97	79 (21.07%)	18 (48.65%)	3.773	0.0002
Pleural thickening	123	119 (31.73%)	4 (10.81%)	2.653	0.0080
Fungal ball	140	138 (36.8%)	2 (5.4%)	3.847	0.0001
Air way only	120	117 (31.2%)	3 (8.11%)	2.949	0.0032
**Microbiological data**					
Sputum culture	294	290 (77.33%)	4 (10.81%)	8.539	<0.0001
BALF culture	101	71 (18.93%)	30 (81.08%)	8.384	<0.0001
Serum GM	64	42 (11.2%)	22 (59.46%)	7.732	<0.0001

BALF GM	75	63 (16.8%)	12 (32.43%)	2.351	0.0187
Aspergillus specific IgG antibody	61	148 (39.46%)	13 (35.13%)	0.515	0.6064
